# First successful percutaneous transhepatic cholangioscopic recanalization of post-hepatectomy biliary atresia with concurrent bile leak

**DOI:** 10.1055/a-2780-6663

**Published:** 2026-03-02

**Authors:** Mengying Zhao, Jingyi Zhang, Jie Zhang, Rongxing Zhou

**Affiliations:** 1 34753 Division of Biliary Surgery, Department of General Surgery, West China Hospital, Sichuan University, Chengdu, China; 2 34753 Research Center for Biliary Diseases, West China Hospital, Sichuan University, Chengdu, China; 3 34753 Department of Medical Ultrasound, West China Hospital, Sichuan University, Chengdu, China

A 58-year-old woman developed biliary complications 1 month after left hemihepatectomy with common bile duct exploration for hepatocellular carcinoma. She presented with abdominal pain, markedly reduced T-tube output (approximately 5 mL/d), and a large postoperative biloma requiring percutaneous drainage (approximately 1,200 mL/d).

Percutaneous transhepatic cholangioscopy (PTCS) confirmed complete biliary occlusion. Direct visualization demonstrated surgical sutures encircling the obstructed segment together with an active bile leak at the same site. PTCS has been established as a reliable technique for direct biliary inspection and therapeutic intervention in complex biliary disorders 1 . The exact etiology of the occlusion could not be definitively determined, but it was presumed to be related to suture-related compression, bile leakage, or both.

**Fig. 1:**
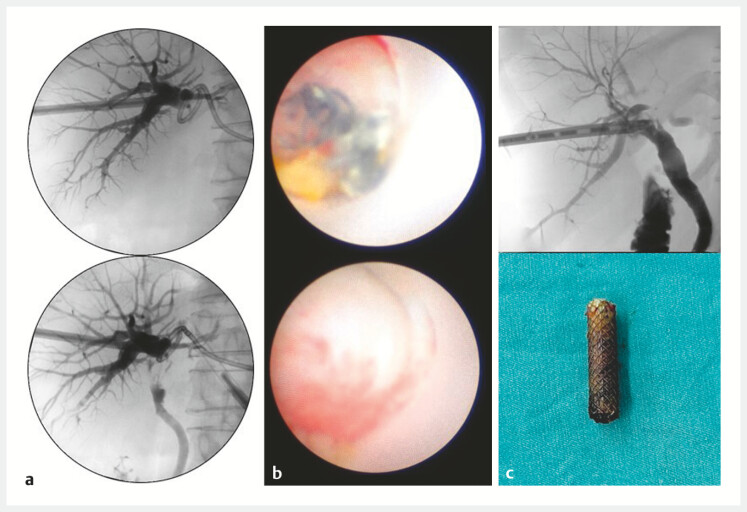
Multimodal imaging and cholangioscopic findings during PTCS-guided recanalization. **a** Pre-intervention percutaneous cholangiography demonstrating complete biliary occlusion at the hepatectomy site with contrast extravasation consistent with an active bile leak. **b** A percutaneous transhepatic cholangioscopic view showing surgical sutures and occlusion. **c** The stent and follow-up cholangiography after stent removal at 8 months confirming restored biliary patency with free contrast flow into the duodenum and complete resolution of the bile leak.

Under real-time ultrasound guidance, combined rigid and flexible choledochoscopy was used to achieve precise recanalization of the occluded bile duct ( Video 1 ). After successful guidewire passage, a fully covered self-expanding metal stent (FCSEMS) was deployed across the occluded segment ( Fig. 1 ). FCSEMS placement has been shown to be effective for benign biliary strictures and bile leaks while allowing safe stent removal 2 3 .

Under real-time ultrasound guidance and direct cholangioscopic visualization, precise puncture of the atretic bile duct segment.Video 1

The patient recovered uneventfully with progressive reduction of biloma output and restoration of internal biliary drainage. At a 8-month follow-up, the stent was removed via PTCS. Follow-up cholangiography demonstrated the complete resolution of the bile leak and a patent biliary tract. Temporary endoscopic or percutaneous stenting has been increasingly emphasized as a minimally invasive alternative to surgical re-exploration for postoperative bile leaks 4 .

Recent reports have also confirmed the feasibility of PTCS-guided recanalization for completely obstructed bile ducts 5 . This case further supports PTCS-guided recanalization combined with temporary FCSEMS placement as an effective minimally invasive strategy for the simultaneous management of post-hepatectomy biliary occlusion and bile leak.

Endoscopy_UCTN_Code_TTT_1AR_2AJ
